# Independently Tunable Fano Resonances Based on the Coupled Hetero-Cavities in a Plasmonic MIM System

**DOI:** 10.3390/ma11091675

**Published:** 2018-09-10

**Authors:** Qiong Wang, Zhengbiao Ouyang, Mi Lin, Qiang Liu

**Affiliations:** 1 THz Technical Research Center of Shenzhen University, Shenzhen University, Shenzhen 518060, China; qwang@szu.edu.cn (Q.W.); linfengas111@szu.edu.cn (M.L.); qliu@szu.edu.cn (Q.L.); 2 College of Electronic Science &Technology, Shenzhen University, Shenzhen 518060, China

**Keywords:** tunable fano resonances, surface plasmon polaritons, coupled cavities, finite element method

## Abstract

In this paper, based on coupled hetero-cavities, multiple Fano resonances are produced and tuned in a plasmonic metal-insulator-metal (MIM) system. The structure comprises a rectangular cavity, a side-coupled waveguide, and an upper-coupled circular cavity with a metal-strip core, used to modulate Fano resonances. Three Fano resonances can be realized, which originate from interference of the cavity modes between the rectangular cavity and the metal-strip-core circular cavity. Due to the different cavity-cavity coupling mechanisms, the three Fano resonances can be divided into two groups, and each group of Fano resonances can be well tuned independently by changing the different cavity parameters, which can allow great flexibility to control multiple Fano resonances in practice. Furthermore, through carefully adjusting the direction angle of the metal-strip core in the circular cavity, the position and lineshape of the Fano resonances can be easily tuned. Notably, reversal asymmetry takes place for one of the Fano resonances. The influence of the direction angle on the figure of merit (FOM) value is also investigated. A maximum FOM of 3436 is obtained. The proposed structure has high transmission, sharp Fano lineshape, and high sensitivity to change in the background refractive index. This research provides effective guidance to tune multiple Fano resonances, which has important applications in nanosensors, filters, modulators, and other related plasmonic devices.

## 1. Introduction

Noble metallic nanostructures that support surface plasmon polaritons (SPPs) have stimulated tremendous research interest due to their special capabilities of overcoming traditional optical diffraction limits, controlling light in the nanoscale domain, and producing extremely strong local electromagnetic fields [1,2,3,4,5]. They provide the possibility for devices with extraordinary properties, high-degree miniaturization, and large-scale integration. As we know, due to electromagnetic wave interactions with the metal surfaces in SPP devices, the transmission characteristics in the devices are closely related to the shape and size of the designed geometry. By now, various plasmonic devices have been proposed, such as optical filters, wavelength division multiplexers, and information modulators [6,7], which open up new opportunities for fabricating plasmonic integrated on-chip systems. 

On the other hand, Fano resonance [8,9,10,11,12,13,14] has emerged as a new research aspect in SPP devices, since it exhibits sharp and asymmetric lineshape in spectra, and small perturbations can induce dramatic intensity variation and wavelength shift. Fano resonance arises from the coherent coupling and interference between a discrete state (or a narrow spectrum) and a continuous state (or a broad spectrum), so that unique spectral patterns can be produced [15,16,17]. Recently, considerable effort has been devoted to research on the tunability of Fano resonance, as it has important applications in nanosensors, filters, slow-light devices, modulators, and so on [18,19,20]. For example, it has been reported that a type of E-shaped plasmonic nanostructure was formed by the interference of the quadrupole resonance modes from a C-shaped metal ring with the dipolar resonance modes from a metal strip, in which the tunability can be easily realized by changing the asymmetry of the geometry [21]. In addition, a compact plasmonic sensor has been designed with a stub and a side-coupled split-ring resonator, and the wavelength position and Fano lineshape can be adjusted by changing the opening direction of the split ring [22]. Additionally, a kind of electrically tunable Fano-type resonance of asymmetric metal-wire pairs has been achieved by controlling the varactor diode loaded on the plasmonic device [23].

For some specific plasmonic structures, multiple Fano resonances can be obtained [24,25], but the independent tunability has rarely been reported. The research on this topic should be given more attention since it will bring great flexibility for fabricating multiple-function devices.

In this paper, based on coupled hetero-cavities, independently tunable Fano resonances are produced and investigated in a plasmonic metal-insulator-metal (MIM) system. This consists of a rectangular cavity, a side-coupled waveguide, and an upper-coupled circular cavity with an angle-tunable metal strip in the center. Through investigating the coupling effect of the two above-mentioned cavities, the mechanisms of Fano resonances are explored. Due to the different cavity-cavity coupling effects, independent tunability can be realized by changing different cavity parameters. Furthermore, the influence of the direction angle of the metal-strip core on Fano resonances is investigated. The figure of merit (FOM) values for different direction angles are also calculated and compared. This research provides an affective measure to produce multiple Fano resonances and realize independent tunability.

## 2. Structure Design

**Figure 1:**
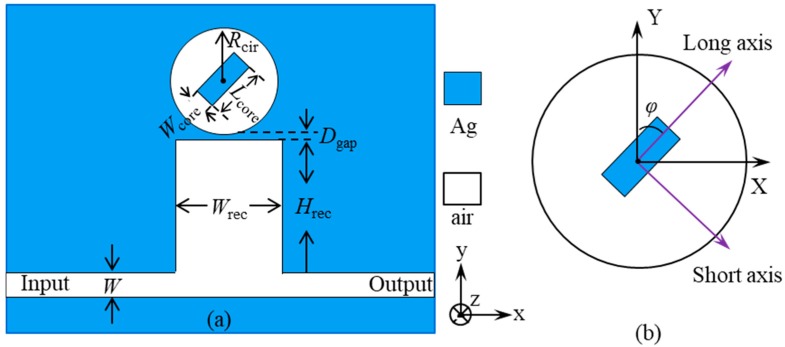
(**a**) Schematic illustration of a two-dimensional plasmonic metal-insulator-metal (MIM) nanosystem consisting of a rectangular cavity coupled with a waveguide, and an upper-coupled circular cavity with an angle-tunable metal strip located at the center. (**b**) In the metal-strip-core circular cavity, the rotation direction of the metal strip is defined by an angle *φ* between the *y*-axis and the long axis of the metal strip.

The X-Y top view of the designed plasmonic nanosystem based on coupled hetero-cavities is shown in Figure 1a. Considering the calculation time, a two-dimensional model is used to demonstrate the characteristics of the structure. It is necessary to emphasize that the MIM structure is chosen because it has the remarkable advantages of long propagation distance, deep-subwavelength field confinement, low bend loss, and easy integration [26,27,28,29]. 

As shown in Figure 1a, a rectangular cavity is connected to a MIM waveguide. The width and height of the rectangular cavity are denoted as *W*_rec_ and *H*_rec_, respectively. The waveguide has a width of *W* = 65 nm. Above the rectangular cavity it has an upper-coupled circular air cavity with a metal-strip core in the center. The radius of the circular cavity is denoted as *R*_cir_. The vertical gap between the circular and rectangular cavities is set as *D*_gap_ = 10 nm. *L*_core_ and *W*_core_ represent the length and width of the metal strip, respectively. In order to modulate Fano resonances, the rotation direction of the metal strip is adjusted. An angle *φ* between the *y*-axis and the long axis of the metal strip is defined, as shown in Figure 1b. The blue and white areas denote the noble metal of silver and air, respectively. For silver, its frequency-dependent complex relative permittivity is characterized by the Drude model [30,31]:

(1) $$\varepsilon_{m}(\omega )=\varepsilon_{\infty }-\frac{\omega_{p}^{2}}{\omega (\omega +i\gamma )}$$

where *ε*_∞_ is the dielectric constant at infinite frequency, *γ* is the electron collision frequency, *ω* is the frequency of the incident light, and *ω_p_* is the bulk plasma frequency. The parameters are *ε*_∞_ = 3.7, *ω_p_* = 1.38 × 10^16^ Hz, and *γ* = 2.73 × 10^13^ Hz. 

In the following section, the transmission characteristics of the coupled hetero-cavity system are numerically simulated in detail using COMSOL software (Version 5.3, Stockholm, Sweden). The structure is divided into a grid of about 5 × 10^4^ small cells. Perfectly matched layers are added around the calculated domain to absorb the electromagnetic waves going out of the structure. A transverse magnetic (TM) wave is launched at the left waveguide. The incident power *P*_in_ and transmitted power *P*_out_ are detected by two power monitors set at the input and output ports, respectively. The total transmission of the coupled system is calculated as *P*_out_/*P*_in_.

## 3. Results and Discussion

### 3.1. The Coupling Mechanism of the Three Fano Resonances

As we know, Fano resonance always exhibits a sharp and asymmetric spectral pattern, which results from the coupling effect between a wide continuous state and a narrow distinct state. In this section, we will investigate the Fano resonance of the coupled hetero-cavity system. In order to explore the mechanisms of Fano resonances, it is necessary to study the rectangular cavity and the metal-strip-core circular cavity separately, and then consider their coupling effect.

**Figure 2:**
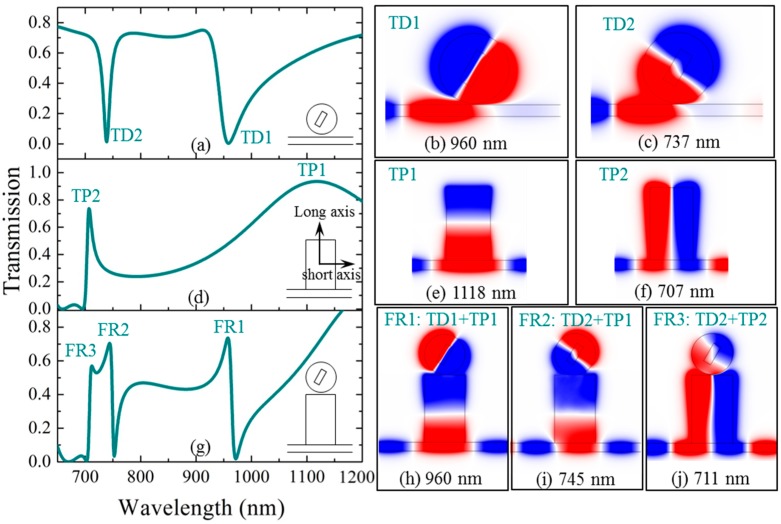
(**a**) The transmission of a metal-strip-core cavity coupled with a waveguide. The *H_z_* magnetic field distributions of the transmission dips at (**b**) TD1, *λ* = 960 nm; (**c**) TD2, *λ* = 737 nm. (**d**) The transmission of a rectangular cavity connected with a waveguide. The *H_z_* magnetic field distributions of the transmission peaks at (**e**) TP1, *λ* = 1118 nm; (**f**) TP2, *λ* = 707 nm. (**g**) The transmission of a coupled system consisting of a waveguide, a rectangular cavity, and a metal-strip-core circular cavity. The *H_z_* magnetic field distributions of the Fano peaks at (**h**) FR1, *λ* = 960 nm; (**i**) FR2, *λ* = 745 nm; (**j**) FR3, *λ* = 711 nm.

Figure 2a shows the transmission of a metal-strip-core circular cavity coupled with a waveguide. The structure is plotted in the inset. The radius of the circular cavity is chosen as *R*_cir_ = 150 nm, and the cavity-waveguide gap is set as *D*_gap_ = 10 nm. For the metal strip of the circular cavity, its width, length, and direction angle are set as *W*_core_ = 60 nm, *L*_core_ = 150 nm, and *φ* = 30°, respectively. It can be seen that the circular cavity keeps a certain distance from the waveguide, thus strong cavity modes can only be excited at particular wavelengths. In Figure 2a, it can be seen that two very narrow transmission dips appear at the wavelengths of *λ* = 960 nm and *λ* = 737 nm, respectively. For convenience, the two modes are named as TD1 and TD2, respectively. Notably, TD1 exhibits an asymmetrical resonance shape. This can be considered as a type of simple Fano resonance that originates from the mode coupling of the metal-strip-core circular cavity and the waveguide. Meanwhile, through adjusting the direction angle *φ* of the metal strip core in the cavity, the coupling effect can be tuned. TD1 and TD2 correspond to two different dipole modes that are anti-symmetric about the long and short axes of the metal strip, respectively, as can be seen from the simulated *H_z_* magnetic field distributions shown in Figure 2b,c.

Figure 2d shows the transmission of a rectangular cavity connecting with a waveguide. The width and height of the rectangular cavity are chosen as *W*_rec_ = 300 nm and *H*_rec_ = 500 nm, respectively. It is necessary to point out that the rectangular cavity is chosen to be large enough, and designed to directly connect with the waveguide, so that the cavity modes can be efficiently and strongly excited at a broad range of wavelengths. As shown in Figure 2d, we can see that two transmission peaks appear at the wavelengths of *λ* = 1118 nm and *λ* = 707 nm, which are named as TP1 and TP2, respectively. It is obvious that mode TP1 has a very wide peak, with a half-high width which is from *λ* = 949 nm to *λ* = 1323 nm. Mode TP2 has a half-high width of 25 nm, which is not as wide as mode TP1. The corresponding *H_z_* magnetic field distributions are given in Figure 2e,f, respectively. Strong cavity modes can be observed, which are anti-symmetric about the short and long axes of the rectangular cavity, respectively.

Figure 2g shows the transmission of the coupled hetero-cavity system. The parameters of the two cavities are the same as those in Figure 2a,d. The cavity-cavity gap is set as *D*_gap_ = 10 nm. We can observe that three resonances with sharp and asymmetric spectral patterns appear at *λ* = 960 nm, *λ* = 745 nm, and *λ* = 711 nm, denoted by FR1, FR2, and FR3, respectively. The lineshapes have the typical characteristic of Fano resonance. The three Fano resonances in Figure 2g can be regarded as the coupling of the cavity modes from the rectangular cavity and the metal-strip-core cavity. In order to further investigate the coupling effects of the Fano resonances, the *H_z_* magnetic field distributions for the three Fano peaks of FR1, FR2, and FR3, are simulated and illustrated in Figure 2h–j. This shows that they originate from three different types of cavity-cavity coupling mechanisms, that is, FR1, FR2, and FR3 are formed by the coupling effects of modes TD1 and TP1, modes TD2 and TP1, and modes TD2 and TP2, respectively, where TD1 and TD2 are the transmission dips of the metal-strip-core circular cavity, and TP1 and TP2 are the transmission peaks of the rectangular cavity.

### 3.2. The Characteristics of Independent Tunability of the Three Fano Resonances

**Figure 3:**
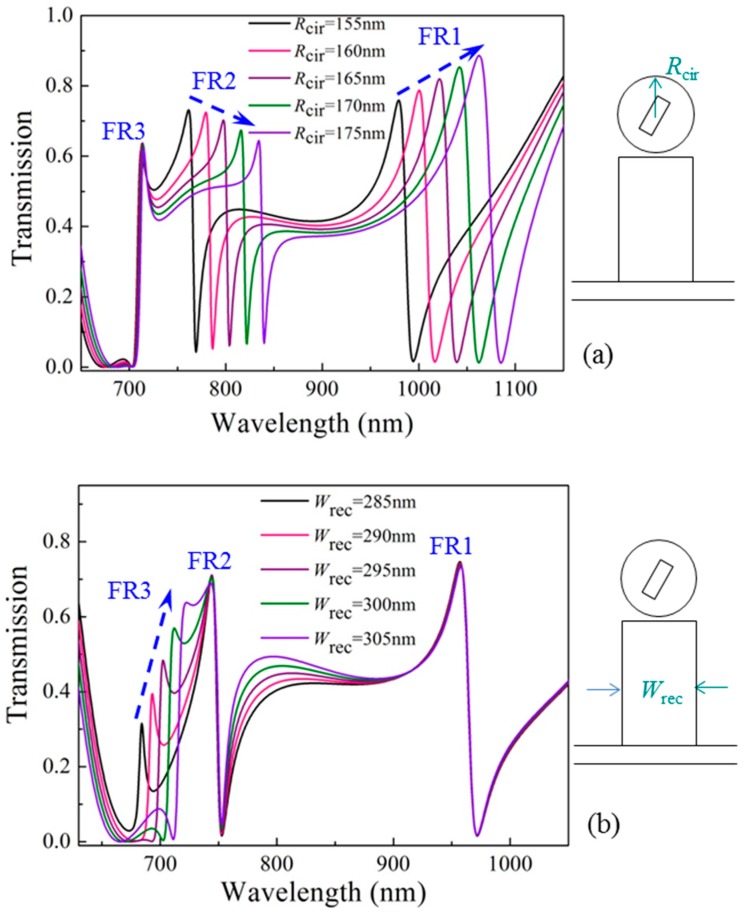
Transmission changes for (**a**) different radii of the circular cavity, *R*_cir_ = 155 nm, 160 nm, 165 nm, 170 nm, 175 nm; (**b**) different widths of the rectangular cavity, *W*_rec_ = 285 nm, 290 nm, 295 nm, 300 nm, 305 nm.

In order to further understand the Fano resonances, the influence of the cavity parameters on transmission is investigated in detail. Figure 3a shows the change of transmission with the radius of the circular cavity increasing from *R*_cir_ = 155 nm to *R*_cir_ = 175 nm. The other parameters remain unchanged. We can see that the Fano peaks of FR1 and FR2 have obvious red shifts, with wavelength increments of Δ*λ* = 83 nm and Δ*λ* = 74 nm, respectively, while the Fano peak of FR3 is nearly unchanged. This proves that the Fano resonances FR1 and FR2 can be simultaneously adjusted by changing the parameter of *R*_cir_, and the Fano resonance FR3 is not affected.

Figure 3b shows the change of transmission with the width of the rectangular cavity increasing from *W*_rec_ = 285 nm to *W*_rec_ = 305 nm. The other parameters remain unchanged. The result is the opposite compared with the above case. The Fano peaks of FR1 and FR2 have almost no change, while the Fano peak of FR3 exhibits a red shift of Δ*λ* = 40 nm in wavelength. This proves that the Fano resonance FR3 can be freely adjusted by changing the parameter of *W*_rec_, and the other two Fano resonances, FR1 and FR2, are not affected.

From the above results, we can find an interesting phenomenon. The three Fano resonances can be divided into two groups. Fano resonances FR1 and FR2 belong to the same group (Group 1), and Fano resonance FR3 belongs to the other group (Group 2). The two groups of Fano resonances can be independently controlled by different cavity parameters. This phenomenon can be understood from the resonances of the rectangular cavity. As shown in Figure 2e,f, the input light is set to be a transverse magnetic (TM) plane wave, thus the resonance mode of the rectangular cavity can be denoted as TM*_mn_*, where *m* and *n* are integers representing the resonant orders in the transverse (*x*-axis) and longitudinal (*y*-axis) directions of the rectangular cavity. The transmission peaks TP1 and TP2 can be regarded as TM_01_ (see Figure 2e) and TM_10_ (see Figure 2f) modes, respectively. For the coupled hetero-cavity system, the *H_z_* field distribution of the rectangular cavity for FR1 or FR2 is TM_01_ (see Figure 2h,i), which is sensitive to the longitudinal parameters, such as the height of the rectangular cavity (*H*_rec_), or the radius of the circular cavity (*R*_cir_). Due to the strong coupling effect existing between the hetero-cavities, the longitudinal resonance of the rectangular cavity is more sensitive to the change of *R*_cir_ than that of *H*_rec_. Therefore, *R*_cir_ is chosen as an example to independently control the Fano resonance FR1 (or FR2). On the other hand, the *H_z_* field distribution of the rectangular cavity for FR3 is TM_10_ (see Figure 2j), which is sensitive to the transverse parameters, such as the width of the rectangular cavity (*W*_rec_).

### 3.3. Tuning Fano Resonances by Changing the Direction Angle of the Metal Strip

**Figure 4:**
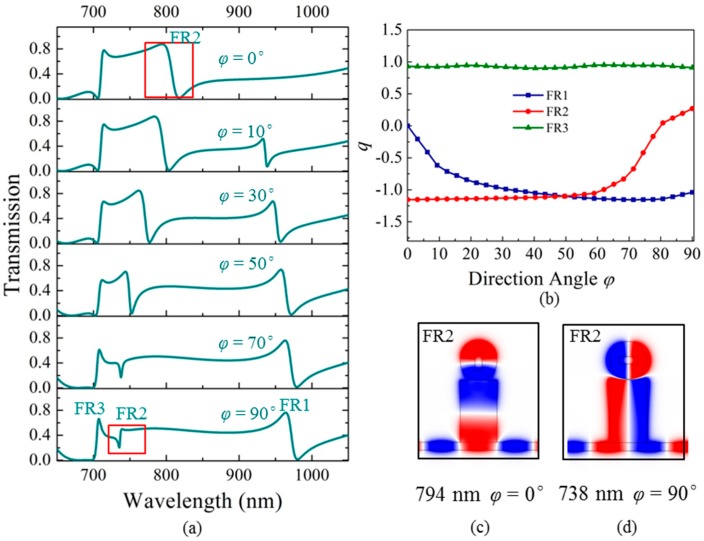
(**a**) Transmissions from changing the direction angle *φ* of the metal strip in the circular cavity with *φ* = 0°, 10°, 30°, 50°, 70°, and 90°. (**b**) The *q* values are calculated for *φ* changing from 0° to 90°. The *H_z_* magnetic field distributions for the Fano peak of FR2 at (**c**) *φ* = 0° and (**d**) *φ* = 90°.

In order to investigate the tunability of the nanostructure, the influence of the direction angle *φ* on Fano resonances is also considered. When *φ* is changed, the field distribution of the circular cavity varies accordingly, which can directly affect the coupling of the hetero-cavities. Thus, the Fano resonances are easily tuned. Figure 4a shows the transmissions with *φ* changing from 0° to 90°. We can see that when *φ* increases, FR1 becomes stronger and FR2 becomes weaker, both accompanied by obvious wavelength shifts, while FR3 has almost no change. The reason for the above difference between FR1, FR2, and FR3, is because *φ* has greater impact on the longitudinal resonance mode TM_01_ of the rectangular cavity (existing in FR1 and FR2) than that of the transverse resonance mode TM_10_ (existing in FR3).

Remarkably, we notice that when *φ* increases to an angle of 90°, the Fano resonance FR2 turns into the reversal Fano asymmetry, as can be seen by comparing the two cases of *φ* = 90° and *φ* = 0°, marked by the two red squares in Figure 4a. Further verification can be obtained by calculating the *q* values from the Fano formula. The transmission can be expressed by the following Fano formula [32],

(2) $$T=T_{\mathrm{Bethe}}+C\frac{{\left(\lambda -\lambda_{\mathrm{Res}}+q\Gamma /2\right)}^{2}}{{\left(\lambda -\lambda_{\mathrm{Res}}\right)}^{2}+{\left(\Gamma /2\right)}^{2}}$$

where *T*_Bethe_ is the direct transmission referred to as Bethe’s contribution, *C* is the non-resonant transmission coefficient, *λ*_Res_ is the resonant wavelength, Γ is the linewidth, and *q* is a dimensionless parameter that describes the asymmetry profile. Using the Origin software, the transmissions of Fano resonances FR1, FR2, and FR3 are fitted with Equation (2). When the fitting error reaches the minimum value, the fitted line expressed by Equation (2) can be determined. Then the variables *T*_Bethe_, *C*, *λ*_res_, *q*, and Γ are fixed. We investigate the dependence of the asymmetry parameter *q* on the direction angle *φ*, as shown in Figure 4b. We can find that for Fano resonance FR2, the *q* value varies from negative (*q* = −1.15) for *φ* = 0° to positive (*q* = 0.27) for *φ* = 90°. This proves that the reversed asymmetry of the Fano resonance is formed. This phenomenon is attributed to the difference in the phase shifts of the cavity modes caused by adjusting the direction angle *φ*. The phase shift is defined as the phase difference between the input and output positions of the rectangular cavity. In Figure 4c,d, the *H_z_* magnetic field distributions for the two cases of *φ* = 0° and *φ* = 90° are shown. For *φ* = 0°, the phase shift of the TM_01_ mode in the rectangular cavity is about 0°, while for *φ* = 90°, FR2 has a blue shift under the influence of field modulation induced by the rotation of the metal strip (from *φ* = 0° to *φ* = 90°), leading to the mode in the rectangular cavity changing from TM_01_ to TM_10_. The corresponding phase shift becomes π. As a consequence, the reversed asymmetry of the Fano resonance is generated. Furthermore, we can see that *q* = 0 is realized at *φ* = 82°, meaning that the resonance exhibits a symmetric profile for this case. This point can be considered as a critical condition to obtain an anti-resonant pattern, and it demonstrates an important tunability option for Fano resonance. The drastic change of the asymmetry parameter can be explored for applications such as optical switches, information modulators, and nonlinear and slow-light devices.

**Figure 5:**
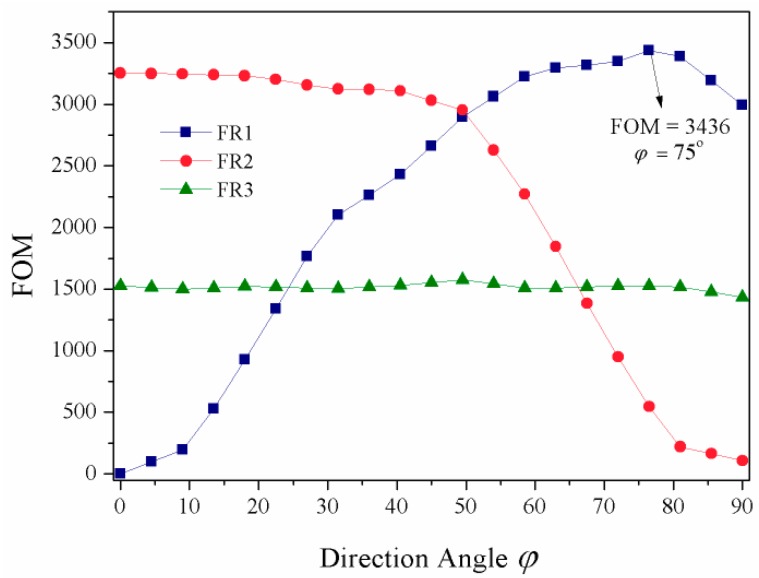
The figure of merit (FOM) values of the Fano resonances FR1, FR2, and FR3 related to the direction angle *φ*.

As we know, sensing performance is one of the most important applications for Fano resonance. Here, the sensitivity characteristic of Fano resonance to the refractive index of the background dielectric material is investigated by calculating the figure of merit (FOM) value [33]. FOM is defined as the maximum of Δ*T*/(*T*Δ*n*) when Δ*n* and *T* are changed. Δ*T*/(*T*Δ*n*) describes the relative transmission variation Δ*T*/*T* at a fixed wavelength induced by the refractive index change Δ*n* of the dielectric material, where *T* denotes the transmission in the proposed structure. As shown in Figure 5, the FOM values of Fano resonances FR1, FR2, and FR3, are calculated when the direction angle *φ* is scanned from 0° to 90°. The other parameters remain unchanged. We can see that the FOM value of FR1 becomes larger with the increase of *φ* at first, and then it reaches the maximum value of 3436 at *φ* = 75° (*λ* = 978 nm at the Fano dip), after that it begins to decrease slightly. The high FOM value contributes to the sharp Fano lineshape in transmission. For FR2, the FOM value decreases with the increase of *φ*. This is because the intensity of the Fano resonance becomes weaker. In contrast, the FOM of FR3 has little change, with the FOM value almost keeping above 1420. Comparing Figure 4b and Figure 5, we can see that a sharp Fano lineshape always has large |*q*|, which is beneficial to obtain a large FOM value for sensing. The proposed structure has high transmission, sharp Fano lineshape, and high sensitivity to the change in background refractive index, which has important applications in nanosensors, filters, modulators, and other related plasmonic devices.

## 4. Conclusions

In summary, a type of coupled hetero-cavity structure is proposed to produce tunable Fano resonances in a plasmonic MIM system. It consists of a rectangular cavity, a side-coupled waveguide, and an upper-coupled circular cavity with a metal-strip core used to modulate Fano resonances. Three Fano resonances can be realized, which originate from the interference of the modes of the rectangular cavity and the metal-strip-core circular cavity. Due to the different cavity-cavity coupling mechanisms, the three Fano resonances can be divided into two groups, and each group of Fano resonances can be well independently tuned by changing the different cavity parameters, which has the advantage of flexibly modulating Fano resonances. Furthermore, through carefully adjusting the direction angle of the metal-strip core in the circular cavity, the position and lineshape of the Fano resonances can be easily tuned. Notably, reversal asymmetry takes place for one of the Fano resonances when the direction angle of the metal strip is changed to be *φ* = 90°. The results show that a maximum FOM value of 3436 is obtained. This research provides effective guidance to produce and tune multiple Fano resonances, which has important applications in nanosensors, filters, slow-light devices, modulators, and other related plasmonic devices.
